# Clarifying the Specificity of Transcranial Pulse Stimulation in Neuromodulatory‐Based Therapeutic Applications

**DOI:** 10.1111/cns.70325

**Published:** 2025-02-28

**Authors:** Onur Alti, Antonino Vallesi

**Affiliations:** ^1^ Department of Neuroscience University of Padova Padova Italy; ^2^ Padova Neuroscience Center University of Padova Padova Italy

1

We would like to address and clarify some critical points regarding the review article by Chen et al. [1], titled: “Transcranial Pulse Stimulation in Alzheimer's Disease”, published in 2024 in the *CNS Neurosci Ther* journal. While we appreciate the authors' efforts in reviewing Alzheimer's related empirical studies using Transcranial Pulse Stimulation (TPS), there appears to be significant ambiguity regarding the neuromodulatory techniques actually used in a considerable portion of the studies cited in that review. Specifically, the review conflates TPS, an ultrasound‐based intervention, with Transcranial Pulsed Current Stimulation (tPCS), a low‐intensity ‘electrical’ brain stimulation technique. This conflation obscures the distinct mechanism of action and potential applications of TPS, leading to confusion for researchers, clinicians and policymakers. In this letter, we clarify these misinterpretations and discuss their implications, while integrating updated insights into TPS mechanisms and principles.

## 2 Mechanism of TPS

TPS is a non‐invasive neuromodulation technique that utilizes focused ultrasound energy to deliver localized brain stimulation. Unlike electrical methods, such as tPCS or Transcranial Direct Current Stimulation (tDCS), TPS leverages the mechanical and acoustic effects of ultrasound, offering distinct mechanisms for inducing neuroplastic changes.

TPS induces neuromodulation via high‐pressure acoustic pulses, which create mechanical stress on neural tissue, prompting neuroplasticity [2]. Unlike tPCS and tDCS, TPS does not use electrodes or electrical current, avoiding skin‐related side effects such as redness or tingling. Its unique properties allow for non‐invasive, focal stimulation with minimal discomfort, offering promise for treating neurological disorders.

## 3 Key Parameters and Safety Constraints of TPS

According to Beisteiner et al. [2], TPS operates with tightly regulated parameters to ensure safety and efficacy:

- Pulse Duration: Approximately 3 microseconds (μs).
- Energy Flux Density: Ranges from 0.2–0.3 mJ mm^2^, with a maximum of 0.25 mJ mm^2^ at 4 Hz.
- Repetition Frequency: Clinically approved range is 1–5 Hz. Although the system can generate frequencies up to 8–10 Hz [3, 4, 5] according to the literature, this exceeds the safety constraints stated by Beisteiner et al. [2] and is not used clinically.
- Spatial‐Peak‐Temporal‐Average Intensity (I_SPTA_): Limited to 0.1 cm^2^.
- Peak Pressure: Up to 25 MegaPascal (MPa), enabling precise acoustic energy delivery without thermal or mechanical tissue harm.
- Maximum Pulses per Treatment: 6000, as defined by unpublished experiments for dose finding and CE approval.

Current evidence suggests that ultrasound neuromodulation is safe, with potential risks being similar for both cortical and deep stimulation [6].

## 4 Examples of Misreporting and Misinterpretations in the Article by Chen et al.

In the discussion section, Chen et al. [1] state: “The frequency of the stimulation is one of the important parameters that significantly affect the TPS effect [original reference #38]. Previous studies have found that random frequency parameters (1–5 Hz) increase functional connectivity in the brain compared to nonrandom and spurious stimuli [#39‐40]. Morales‐Quezada et al. [#41] also demonstrated that a random frequency of TPS between 6 and 10 Hz also led to an increase in brain functional connectivity, as shown by the facilitation of electroencephalogram spectral power and connectivity measurements”. This passage references the frequency range of 6–10 Hz, which is outside the feasible limits for ultrasound‐based TPS, as TPS devices are typically limited to upper‐bound of approximately 5 Hz^2^. In fact, the studies by Saavedra et al. [7] [orig. #39] and Morales‐Quezada et al. [8] [orig. #40] investigate the effects of tPCS, and not TPS, on brain connectivity. This misattribution also confuses the putative neuromodulatory mechanisms of TPS, which specifically operates through acoustic energy rather than electrical currents.

Another example within the article [1] reads: “In a study of TPS for lower limb spasticity in children with cerebral palsy, only mild skin redness (0.05%) at the electrode site was reported [#46]. The safety of TPS in the treatment of Parkinson's disease has been studied, and no adverse events have been observed [#47]. TPS is a low‐intensity transcranial electrical stimulation, and mild side effects similar to tDCS may occur during treatment, such as itching, tingling, burning, and transient redness [#48]”. In this passage, the review article [1] inaccurately associates TPS with applications in cerebral palsy for which, to our knowledge, TPS has not been studied yet. Additionally, the reference to “itching, tingling, burning, and transient redness” are possible adverse effects of transcranial electrical stimulation, as seen in studies using tDCS and tPCS, not of ultrasound‐based TPS. Moreover, the only published studies on TPS with Parkinson's disease patients to date are those by Osou et al. [9] and Manganotti et al. [10], which did not report such side effects. This mischaracterization not only creates a misunderstanding of the safety profile of TPS, but also further obscures its implementation and underlying mechanisms, as ultrasound‐based TPS does not rely on skin‐contact electrodes.

Other mis‐cited articles in the review include Jaberzadeh et al. (original ref. #56), Thibaut et al. (#57), Singh et al. (#58), Vasquez et al. (#59), Morales‐Quezada et al. (#62), Ruhnau et al. (#63), and Zarifkar et al. (#66), all of which pertain to electrical stimulation, specifically tPCS or related transcranial electrical neuromodulation techniques, rather than ultrasound‐based TPS.

## 5 Broader Implications for the Field of Neuromodulation and Recommendations

These misinterpretations actually highlight a larger issue in neuromodulatory research literature: the conflation of heterogeneous non‐invasive brain stimulation (NIBS) techniques. With the growing diversity of techniques, such as TPS, tDCS, and tPCS, each with its own unique procedures, mechanisms of action, and clinical applications, it is essential for researchers to distinguish them unambiguously.

We propose the following recommendations to address this issue:

1. Standardized Terminology: Researchers should differentiate between “pulse” in ultrasound‐based TPS and “pulsed” in electrical stimulation techniques like tPCS. Consensus definitions, akin to those established for Transcranial Magnetic Stimulation (TMS) [11, 12], would aid clarity.
2. Rigorous Peer Review: Journals should emphasize methodological precision and verify that cited studies align with the discussed intervention.
3. Educational Initiatives: Workshops, reviews, and conference presentations should clarify the distinctions between NIBS modalities to reduce confusion and improve understanding.

In conclusion, we appreciate Chen and colleagues' contribution [1] to the discussion on TPS in Alzheimer's disease. However, we urge the scientific community to consider these points of clarification to ensure unbiased research that could accurately contribute to the growing body of literature in this field. Addressing these issues will not only facilitate the progression of NIBS research but also improve clinical outcomes by providing healthcare professionals with reliable information on these interventions. A consensus‐based approach could help prevent future misinterpretations and support the accurate accumulation of evidence for the mechanism of action and therapeutic potential of each unique technique.

## Author Contributions

**Onur Alti, Antonino Vallesi:** conceptualization. **Onur Alti:** writing – original draft. **Onur Alti, Antonino Vallesi:** writing – review and editing. **Antonino Vallesi:** supervision.

## Conflicts of Interest

The authors declare no conflicts of interest.

## Data Availability

The data that support the findings of this study are available on request from the corresponding author. The data are not publicly available due to privacy or ethical restrictions.
